# Detecting gene-gene interactions in prostate disease in African American men

**DOI:** 10.1186/1750-9378-6-S2-S1

**Published:** 2011-09-23

**Authors:** R Renee Reams, Krishna Rani Kalari, Honghe Wang, Folakemi T Odedina, Karam FA Soliman, Clayton Yates

**Affiliations:** 1 College of Pharmacy and Pharmaceutical Sciences, Florida A&M University, Tallahassee, Florida, USA; 2 Department of Medical Informatics, Mayo Clinic College of Medicine, Rochester, MN, USA; 3 Dept of Biology and Center for Cancer Research, Tuskegee University, Tuskegee, Alabama, USA; 4 College of Pharmacy, University of Florida, Gainesville, Florida, USA; 5 Prostate Disease Center, Department of Urology, University of Florida, Gainesville, Florida, USA

## Abstract

**Background:**

The most common male malignancy in the United States is prostate cancer; however its rate of occurrence varies significantly among ethnic groups. In a previous cDNA microarray study on CaP tumors from African American (AA) and Caucasian (CA) patients, we identified 97 candidate genes that exhibited *opposite* gene expression polarity with respect to race groups; genes up-regulated in AA were simultaneously down-regulated in CA.

**Purpose:**

The purpose of this study was to narrow the 97 member gene list, to a smaller number of genes in order to focus studies on a limited number of genes/SNPs that might explain prostate cancer disparity in African Americans.

**Methods:**

We performed genotype-phenotype, SNP and expression transcript levels correlations using HapMap Yoruba population with 85 of our 97 prostate candidate genes using SCAN database.

**Results:**

Findings revealed an association of SNPs surrounding ABCD3 gene with basal gene expression of RanGAP1 is important in prostate tumors in AA. Hence, to confirm our results in clinical biospecimen, we monitored expression of ABCD3 in a novel panel of African American and Caucasian prostate cancer paired cell lines. The LNCaP, C4-2B showed 2-fold increase; MDA-2PC-2B cell line**,** derived from AA, showed highest fold-change, 10-fold. The EGFR over expressing **DU-145 WT** cell line exhibited a 4-fold increase in expression relative to non transfected **DU-145** prostate cell lines. Furthermore, Ingenuity Network analysis implicated our AA prostate candidate genes are involved in three network hubs, ERK, MapK and NFkB pathways.

**Conclusions:**

Taken together, these findings are intriguing because other members of the ABC gene family, namely, ABCC3, ABCD1, and ABCD2 have been shown to confer chemoresistance in certain cancer types. Equally important, is the fact that activation of the MapK/ERK pathway via EGFR stimulation is vital for increased transcription of numerous cancer related genes. It is especially noteworthy that overexpression of EGFR has been widely observed in AA prostate tumors. Collectively our findings lead us to think that a novel signaling cascade, through which increased aggressiveness and chemoresistance is achieved, may explain prostate cancer health disparity in AA males and the nature of aggressive CaP tumors in general.

## Introduction

Prostate cancer (CaP) is the second leading cause of cancer-related death among all men in the United States. However, incidence and mortality rates for this disease vary substantially among geographic areas and ethnic groups. Most notably African American men (AA) in the United States have the highest risk (19%) of developing prostate cancer, and due to the development of more aggressive disease, they have more than twice the mortality rate observed for other racial and ethnic groups [1]. The explanation for these differences is still unknown; however proposed explanations include genetic factors, dietary factors, behavioral factors, biological tumor aggressiveness, socio-economic factors and gene-environment interaction [2-35]. While AA race/ethnicity is one of the three primary non-modifiable risk factors confirmed for CaP, there are only a few published cDNA microarray studies [36-38] that have focused on gene expression differences in AA tumors compared to CA in an attempt to understand prostate cancer health disparity. Previously we identified 97 genes differentially expressed in AA prostate tumors. To narrow down this number of genes, we utilized advance bioinformatics methods. In the present study we performed genotype-phenotype or SNP and expression transcript level correlations of HapMap lymphoblastoid cell lines from Yoruba population to the 97 prostate candidate genes in AA, in an attempt to ferret out genetic variants associated with AA population. In addition, we used Ingenuity pathway analysis to calculate the probability of finding our set of candidate genes within a given pathway(s) to establish probable signal transduction mechanisms.

## Methods

### Microarray prostate candidate gene list for AA tumors

The gene list used in this study was obtained from our previously published cDNA microarray study [36].

### SCAN database analysis to look for gene-gene interactions

SCAN is a large-scale database of genetics and genomics data associated to a web-interface and a set of methods and algorithms that can be used for mining the data in it (http://www.scandb.org/newinterface/about.html). Information on the relationship between SNPs and expression transcript levels (eQTLs) that is served by SCAN comes from a series of publications describing studies characterizing eQTLs in lymphoblastoid cell lines from HapMaP Caucasian (CEU) and Yoruba (YRI) samples for which transcript levels have been assayed using the Affymetrix Human Exon 1.0 ST Array [39-44].

The SCAN database contains two categories of SNP annotations: (1) Physical-based annotation or SNPs categorized according to their position relative to genes (intronic, antigenic, etc.) and according to linkage disequilibrium (LD) patterns (an intergenic SNP can be annotated to a gene if it is in LD with variation in the gene). (2) Functional annotation where SNPs are classified according to their effects on expression levels, i.e. whether they are eQTLs for that gene. Information on physical, functional and LD annotation served on the SCAN database comes directly from public resources, including HapMap (release 23a), NCBI (dbSNP 129), or is information created by using data downloaded from these public resources. In SCAN database, genotype data for the YRI samples was obtained from HapMap project (http://www.hapmap.org). Genotype and gene annotations were obtained from NCBI, dbSNP 129.

We uploaded appropriate gene identifiers for our prostate candidate genes and queried for SNPs that are significantly associated with expression of prostate candidate genes in Yoruba (YRI) population in lymphoblastoid cell lines. SCAN genetic and genomic data for the Yoruba Population in Ibaden, Nigeria, Africa was used because of close ancestral ties of Nigerians to African Americans. The SCAN analysis output reports a list of SNPs in gene(s) that predict expression quantitative trait loci found in mRNA profiles from YRI with p-values less than 0.0001.

### Hugo gene symbols

To enter a list of genes into SCAN, it is first necessary to use the HUGO (Human Genome Organization) gene symbol - the unique gene name and symbol given to each human gene by The HUGO Gene Nomenclature Committee (HGNC). From our 97 gene list, we were able to obtain HUGO gene symbols for 85 of the 97 genes (hereafter referred to as 85/97).

### Ingenuity pathway analysis

Ingenuity software (http://www.ingenuity.com/) was used for pathway analysis. Ingenuity software calculates p-value for the probability of finding a set of genes within a given pathway. Fisher's exact test was used to calculate the p-values associated with finding 536 prostate genes obtained during this study (which includes 85/97 candidate genes, from previous differential expression study [36] and 451 candidate genes that are corresponding to *Cis-* regulatory SNPs, that are significantly associated with the 85 candidate genes) within a annotated network from Ingenuity Knowledge Base.

### RT-PCR validation in novel panel of prostate cancer lines

RT-PCR assay was done using a 7500 FAST Real-Time ABI System.

Briefly, total RNA from each cultured prostate cell line was extracted, separately, with RNAzol B (Tel-Test Inc., Friedswood, Tx) according to the manufacturer’s protocol and quantified with Nucleic Acid Quantitation Kit (NBI, Plymouth, MN). Total RNA (1 ug) was reverse transcribed into cDNA with RT^2^ First strand Kit (SABiosciences/A Qiagen Company)) and 1.10 of the reverse-transcribed product from each sample was used for PCR to amplify ABCD3 gene, using a RT^2^ qPCR Primer Assay for Human ABCD3 (SABiosciences/A Qiagen Company) The expression of GAPDH was used as an internal control/housekeeping gene. Experimental conditions for the ABCD3 gene was optimized to analyze the amplified product in the linear range of amplification by adjusting amplification cycles for each set of primers. The expected band size (bp) size of the PCR product was 83, as described by vendor (SABiosciences).

### Novel prostate cancer cell lines: description

Non-malignant (RC-77N/E) and malignant (RC-77T/E) prostate cells were derived from an African American prostate cancer patient and both are androgen sensitive [45]. RC-77N/E cells were isolated from pathological normal cells, while RC-77T/E were derived from stage T3 tumor. Both cell lines are cultured in Keratinocyte Serum-Free Medium (KGM) Life Technologies, Gaithersburg, Md., USA), supplemented with bovine pituitary extract (BPE), recombinant epidermal growth factor (rEGF), 1% (v/v) penicillin-streptomycin-neomycin (PSN) antibiotic mixtures and 1% (v/v) amphotericin B (KGM) (Life Technologies, Gaithersburg, MD, USA). MDA-2PC-2B, also derived from an African American patient are androgen dependent, metastatic and are cultured in F12 K medium. DU-145, a cell line originally derived from a brain metastasis of a human prostate adenocarcinoma [46] retains the androgen independence of the original tumor and does not express a functional AR [47]. This cell line has both LHRH-R and epidermal growth factor receptors (EGFR) and produces the EGFR ligands, transforming growth factor α (TGF-α) and EGF [48,49]. Utilizing established protocols, DU-145 cells were transfected by retroviral-containing EGFR constructs [50]. The wild-type (WT) EGFR construct is a full-length cDNA derived from a placental cDNA library. Cells expressing WT EGFR at levels that escape downregulation demonstrate enhanced invasiveness in vitro [51]. LNCaP cells were derived from a lymph node metastasis [52]. The Caucasian LNCaP, C42-B prostate cancer cell lines were maintained in T-medium as previously described.

## Results

### Origin of the 97 prostate candidate genes

To detect for gene-gene interactions in AA prostate tumors in African American males, we used a cDNA microarray gene list obtained from a pilot project cDNA microarray comparison study of prostate tumor gene expression in AA and CA [36]. To obtain differentially expressed gene or the gene list, we used four snap frozen tumors and four snap frozen non-tumor matched controls, each, from AA and CA . All tumors had a Gleason score of six. Gene expression profiles were measured for each of the micro dissected CaP tumor samples using Affymetrix U133A human arrays as described in [36]. Each of the 8 prostate tumors and 8 matched controls underwent single hybridization and was arrayed individually (i.e. samples were not pooled). Data from the micro array CEL files were uploaded to R-Bioconductor for analysis [53]. We paired normal AA tissue to tumor AA and paired normal CA to tumor CA to generate for case paired t-tests for each race group; gene lists of differentially expressed genes in AA Tumor vs. AA controls and of CA Tumor vs. CA controls were generated. We looked for differentially expressed genes that met the filtering criteria of a 4.0-fold change and a p< 0.0001. Neither the comparison of AA tumor to AA controls nor the comparison CA tumors to CA controls yielded genes that met both parts of our filtering criteria. However, when we looked at the ratio of CA tumor/CA normal to AA tumor/AA normal (case-matched ratios-race group tests for specific expression trends) we found 97 statistically significant, differentially expressed genes with 4-fold or greater fold change and p<0.0001. It was necessary to ratio the ratios to control for the high degree of genetic variation in AA tumor and AA non-tumor samples.

### Scan database SNP and expression transcript level association results

After uploading our 85/97 prostate candidate genes with appropriate HUGO gene symbols and querying for SNPs in our 85/97 significantly associated with SNPs in the HapMap Yoruba (YRI) population; approximately, 26527 genotype-phenotype associations were obtained with a p-value < 10^-3^, of which 17542/26527 associations had a p-value <10^-4^ ( data not shown)

**Figure 1:**
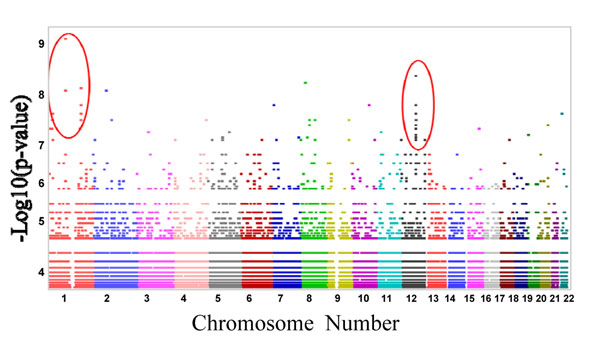
GWAS Plot of Gene-Gene Associations illustrates p-values (shown on y-axis) of SNP for gene variants found on chromosomes 1-22(x-axis). The x-axis shows Chromosomes 1 thru 22. Each dot represents gene variants or SNPs. In the circled dots to the extreme left positioned above Chromosome 1 (x-axis), the dot with the highest p-value represents an ABCD3 gene variant with a defined rs# that is strongly interacting with RANGAP1 to influence CaP tumors in African American. All of the dots in the circle reveal an association of SNPs surrounding ABCD3 gene with basal gene expression of RanGAP1. This variation in gene expression RanGAP1 might be influenced by the SNPs in ABCD3 Similarly in the circled points to the far right, positioned above chromosome 12 ( x-axis), the dot with the highest p-value represents the TMTC2 gene variant with a defined rs#, that strongly interacts with STXBP2. All the dots in the circle represent an association of SNPs surrounding TMTC2 gene with basal gene expression of STXBP2.

SNPs and expression transcript levels (eQTL) associations results identified two gene-gene associations. Association results in lymphoblastoid cell lines showed that expression of RanGAP1 gene which is a key regulator of the RAN GTP/GDP cycle, located on chromosome 22, may be involved with several SNPs in ABCD3 gene which is ATP-Binding cassette, subfamily member that is located on chromosome 1 (Shown as encircled dots on far left in Figure 1). In addition, expression of STXBP2 gene which is a syntaxin-binding protein that is located on chromosome 19 may be involved with a region on chromosome 12. The chromosome 12 region consists of transmembrane and tetratricopeptide repeat containing 2 (TMTC2) gene that is approximately 400kb away from the region where STXBP2 gene is associated (shown as encircled dots on far right of Figure 1). Genome-wide results also showed that there were 1167 *cis* interactions (where expression gene and SNP are located on the same chromosome) out of 26527 associations with a p-value < 10^-3^. Most of the *cis*-regulatory associations were found in protein coding regions.

### CaP candidate genes found in ERK, MapK, NFKB pathways

**Figure 2:**
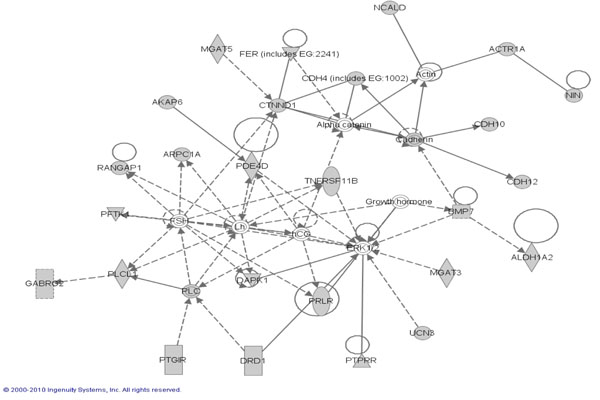
Ingenuity Pathway Analysis identified *ERK* as the Top network signaling Hub where the shaded shapes indicate the candidate genes from the present study. *RANGAP1* is found in the *ERK* pathway. Dotted line indicates an indirect cellular interaction and solid lines indicate a physical interaction between genes. Genes are identified with their HUGO symbol. Dotted line indicates an indirect cellular interaction and solid lines indicate a physical interaction (acts on or inhibits) between genes. Different shapes (diamond, circle or rectangles) of the nodes represent functional classification of the genes.

**Figure 3:**
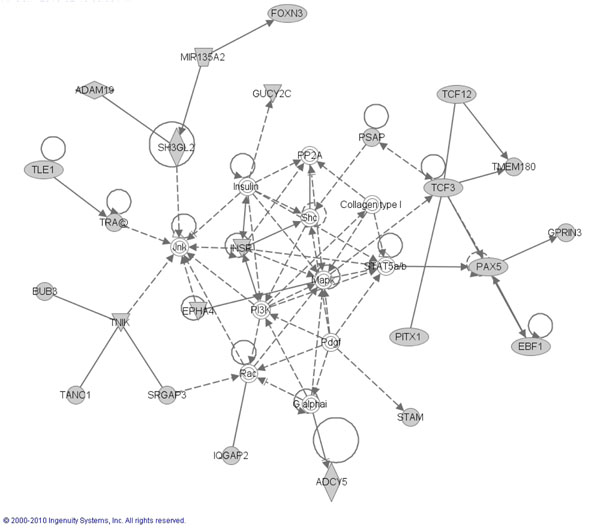
Ingenuity Pathway Analysis identified *MapK* as second Top network signaling Hub where the shaded shapes indicate the candidate genes from current study. Dotted line indicates an indirect cellular interaction and solid lines indicate a physical interaction between genes. Molecules are identified with their HUGO symbol. Different shapes (diamond, circle or rectangles) of the nodes represent functional classification of the genes shown.

**Figure 4:**
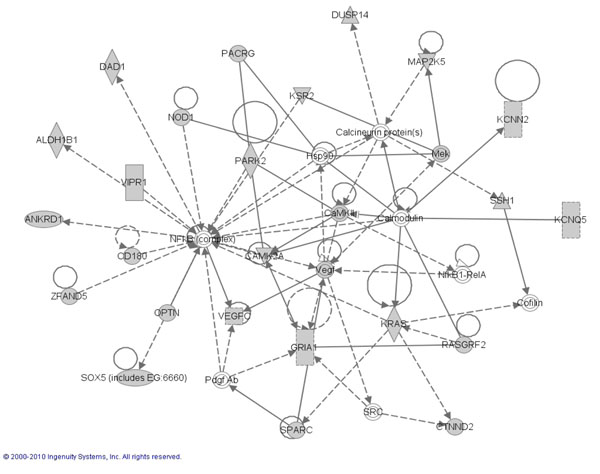
Ingenuity Pathway Analysis identified *NFKB* as the Top Network signaling Hub where the shaded shapes indicate the candidate genes from the present study. Dotted line indicates an indirect cellular interaction and solid lines indicate a physical interaction (.I.e. binding) between genes. Molecules are identified with their HUGO symbol. Different shapes (diamond, circle or rectangles) of the nodes represent functional classification of the genes shown.

Pathway analysis was performed in an attempt to define biological relationships among candidate genes identified during our study using the genes that are involved with the downstream effects of SNPs along with the 85/97 candidate prostate cancer genes as described in methods. Ingenuity Pathway Analysis (IPA) was used to perform the pathway analysis. This software consists of a curated database and several analysis tools to determine the probability of finding a set of genes within annotated pathway or network annotation. Results showed a high probability for finding our candidate genes in three network hubs centered on ERK, MAPK and NFkB pathways (Shown in Figures 2, 3 and 4, respectively). These “top” three networks with p values of <0.05 based on Fisher’s exact test were associated with genetic disorder, cellular development, cell death and cell signaling. Direct interactions between the genes in the network pathways are indicated by solid lines and indirect relationships are indicated by dashed lines. The shaded genes represent our 536 candidate genes identified in our association study as described in method section (85 candidate genes + 451 cis-regulatory genes that are associated with the 85 candidate genes). Diamond shapes represent enzymes, oval represent transcription regulators. Squares: cytokines and triangles: kinases. (For full explanation of shapes and the functional classification they represent go to **http://www.springerimages.com/Images/LifeSciences/1-10.1007_s12014-010-9053-0-2),**

### ABCD3 gene highly expressed in aa metastatic prostate cancer lines

**Figure 5:**
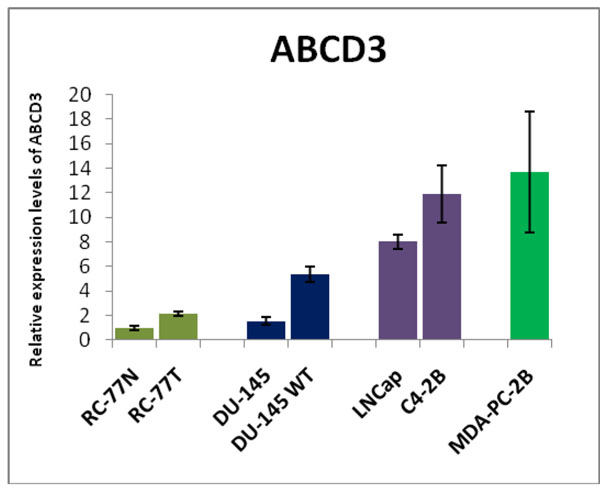
Expression of ABCD3 in panel of paired prostate cancer cell lines. (A) qRT-PCR of non-malignant African American RC-77N/E was compared to malignant RC-77T/E cells, DU-145 was compared to DU-145 WT (EGFR overexpressing), LnCaP was compared to C4-2B, and all samples were compared to African American MDA-PC-2b cells. Results shown is representative of experiments performed in triplicate.

Our association study of 85 candidate genes with genome-wide SNPs in HapMap YRI lymphoblastoid cell lines has revealed an association of SNPs surrounding ABCD3 gene with basal gene expression of RanGAP1 using data obtained from SCAN database (Figure 1). This variation in expression levels of RanGAP1 might be influenced by the SNPs in ABCD3. To confirm our results obtained during the association study, we tested ABCD3 expression in clinically relevant cell lines. Verification of ABCD3 in novel AA/CA prostate cancer cell lines revealed an increase in expression with increased metastasis across a novel panel of African American and Caucasian prostate cancer paired cell lines (Figure 5). The malignant **RC-77T/E** cells isolated from AA showed 2-fold increased expression compared to non-malignant RC-77N/E matched pair (Figure 5). The metastatic, androgen dependent **MDA-2PC-2B cell line** derived from AA ) exhibited a 10-fold ABCD3 expression (Figure 5). Previously we have demonstrated that DU-145 WT (EGFR overexpressing) cells exhibit increased invasiveness and metastasis both in vitro and in vivo [49]. Therefore, we examined ABCD3 gene expression in the DU-145 WT cell and in non-transfected DU-145 cells. DU-145 WT cells showed a 4-fold increase in expression relative to DU-145 prostate cell lines (Figure 5). A similar pattern of expression was observed in the androgen independent metastatic C4-2B cells derived from Caucasian androgen dependent LNCaP cells, thus providing firm evidence of increased ABCD3 gene expression with increased prostate cancer progression in AA tumors (Figure 5).

## Discussion/conclusion

To address the underlying genetic cause of prostate cancer burden in African Americans, we previously obtained a cohort of normal tumor paired samples from African American and Caucasian men, and looked for differential gene expression within each group. Utilizing a strict filtering criteria, we observed over 97 differentially expressed genes in our African American vs. Caucasian sample set [36]. To further narrow the list to genes of utmost importance in prostate disease onset, we used a genome-wide association approach that allowed us to rapidly scan for SNPs in the genome of healthy Yorubai population (Yorubai from Ibaden, Nigeria) that might possibly be associated with our 85/97 prostate candidate genes. We utilized the HapMap database of YBI, Nigerian lymphoblastoid cell lines, since there are a limited number of SNP databases containing African American patients. Most importantly the Yorubai population rather than the Caucasian (CEU) population was utilized because the close ancestral ties [54] between West African Africans and African Americans would increase our chances of finding similar genetic variants associated with prostate disease in men of African descent (Nigerians and African Americans). Approximately 536 genes were identified in our association study as described in methods section (85 candidate genes + 451 cis-regulatory genes) that are associated with the 85/97 candidate genes. Herein we report that a significant number of direct gene-gene interactions were found, however the most significant interactions were observed on chromosome 1 and chromosome 12. Given the importance of these chromosomes in prostate disease, it was appropriate to investigate ABCD3 gene involvement in African American prostate cancer. Our analysis of gene-gene interactions on chromosome 1 and 12 revealed that ABCD3/RanGAP1 and STXBP and TMTC2 gene showed the strongest associations. Of these the ABCD3/RanGAP1 genes were predicted to have the most significant interactions. As such neither ABCD3 or RanGAP1 expression has been implicated in prostate cancer, therefore we chose to focus on ABCD3. The **A**TP-**b**inding **c**assette genes consist of various subfamilies, are typically expressed in both normal and cancer cells. Their functions have been implicated in acquired Multidrug Resistance, MDR, in cancer cell lines. For example, recent reports have shown that MCF-7/AdVp3000 cells that were derived by selection for growth in the presence of doxorubicin, exhibit a 459-fold overexpression of ABCC3 relative to the parental cell line [55]. A similar situation in prostate cancer cell lines has been observed as MDR1/Pgp/ABCB1 and multidrug resistance-associated protein-1(MRP1/ABCC1), with the half ABC transporter, breast cancer resistance protein BCRP/ABCG2, is able to selectively isolate the putative prostate stem cells from the prostate tissue microenvironment through constitutive efflux of androgen and protects the putative tumor stem cells from androgen deprivation, hypoxia, or adjuvant chemotherapy [56]. These findings are supported by unpublished data, from Yates laboratory, that suggest that fluorescence-activated cell sorting, FACS isolation of the (SP) cells, selective for functional ABC transporter pumps, have higher in vivo tumorigenicity compared to other cell surface markers (unpublished data). Thus, it is possible that ABCD3 could possibly contribute to aggressive prostate cancer.

Multiple ABC family genes have been implicated to play a role in chemoresistance and progression of prostate and breast cancer, however given the large number of family subtypes only a few have been associated with prostate cancer progression to aggressive disease. Since ABCD3 gene expression has not been identified previously in prostate cancer, we verified this in commonly utilized prostate cancer cell lines, as well as in a normal and primary tumor cell line pair derived from an African American prostate cancer patient [45,57]. As in prostate patient samples, ABCD3 was consistently overexpressed in the RC-77N/E/RC-77T/E, LNCaP/C4-2B models, with MDA-PC-2B cells exhibiting the highest expression (10-fold). This data is consistent with the expression of other members of the ABC gene family that have been implicated in prostate cancer, and serves as proof-of-principle evidence that ABCD3 overexpression is indeed correlated with prostate cancer progression. That MDA-2PC-2B cells exhibited the highest expression levels, further implicates a role for ABCD3 in African American prostate cancers.

To further substantiate the link of ABCD3 with other cell signaling molecules that contribute to prostate cancer, we utilized an indirect in silico ingenuity pathway analysis. ABCD3, showed a high probability of being found within three growth factor initiated network hubs involving ERK, MAPK and NFkB proteins. The ERK MAPK has been implicated in a number of pathophysiological events including androgen receptor signaling [58] and the epithelial-to-mesenchymal (EMT) [59] that occurs as cancer cells acquire the property to metastasize. That we observed a 4 fold increase in ABCD3 expression in an EGFR overexpressing DU-145 WT cell line compared to non-transfected DU-145 cells, highlights a putative novel regulator of ABCD3. EGFR is overexpressed in African American Prostate patients [60] and a robust activator of MAPK ERK in normal and cancer cell [61]. Furthermore overexpression is sufficient to increase proliferation, invasion related EMT, and metastasis [49,62,63]. Thus, it appears that ABDC3 is a novel prostate cancer associated gene, that could, in part, be regulated by EGFR signaling.

Although further studies to investigate the ABCD3/RanGAP1 relationship need to be conducted, our results clearly illustrate the utility of high density SNP analysis, in conjunction with appropriate cell lines that represent the clinical conditions, to identify regulatory genes in prostate cancer. Although a more in-depth analysis of the exact role of ABCD3 in such events as cell proliferation, and chemoresistance is warranted and are underway. The results of this study provide a rationale for use of DU-145/DU-145WT and MDA-PC-2B as culture models to study molecular mechanisms associated with the health disparity in African American prostate cancer patients.

## Limitation of study

The 97 member candidate gene list was derived from a limited number of prostate tumor samples and matched control from AA and CA. Nonetheless, approximately, 26527 genotype-phenotype associations were obtained with a p-value < 10^-3^, of which 17542/26527 associations has a p-value <10^-4^. The associations with the highest P-values showed two distinct association; one of which has led us to think that the ABC gene family plays an important role in prostate cancer aggressiveness and chemoresistance.

## Competing interests

The authors do not have any competing interests to declare.

## Authors’ contributions

All authors read and approved the final manuscript.

RRR identified the 97 prostate candidate genes in a previous study; RRR was responsible for the GWAS study design, manuscript preparation, editing & critical revisions of the manuscript. KRK conducted the GWAS study and ingenuity pathway analysis studies and contributed to manuscript preparation. HW conducted RT-PCR experiments using novel panel of prostate cell lines contributed by CY. CY established novel panel of prostate cell lines; contributed their use for this study (in his laboratory) and provided critical revisions and pertinent scientific discussions of his published and unpublished data that linked ABCD3 gene expression in metastatic cell lines to being regulated in part by EGFR. FTO. and KFAS participated in scientific discussions, proofing of the manuscript and brought together CY and RRR as collaborators.
